# Protective effects of *Passiflora edulis* Sims peel fermentation polysaccharide against UVB-induced photodamage via antioxidant and anti-inflammatory activities

**DOI:** 10.1186/s40643-026-01075-8

**Published:** 2026-06-03

**Authors:** Jiaxuan Fang, Yifan Fang, Bingbing Fu, Zixin Song, Jianfei Zhao, Qianru Sun, Meng Li, Changtao Wang, Dongdong Wang

**Affiliations:** School of Light Industry Science and Engineering, Beijing Technology & Business University, 11 Fucheng Road, Haidian District, Beijing, 100048 China

**Keywords:** *Passiflora edulis* Sims peel, Fermented polysaccharides, UVB photoprotection, Skin barrier, Oxidative stress

## Abstract

Ultraviolet B (UVB) radiation is the primary cause of photodamage to the skin, triggering the oxidative stress, mitochondrial dysfunction, and impaired barrier integrity. We extracted polysaccharides from *Passiflora edulis* Sims peel via microbial fermentation (PP-FP) and characterized their structural properties using fourier transform infrared spectroscopy (FTIR), scanning electron microscopy (SEM), and molecular weight analyses. Photoprotective effects were evaluated in UVB- irradiated HaCaT cells and BALB/c mice dorsal skin by measuring of oxidative stress markers, mitochondrial function, DNA damage, and skin barrier proteins. PP-FP contains a furanose ring structure, exhibits an irregular, loosely layered morphology with attached filamentous and porous structures, and shows a number-average molecular weight (Mn) of 3.519 kDa and a weight-average molecular weight (Mw) of 6.66 kDa. In UVB-irradiated HaCaT cells, PP-FP (500 µg/mL) significantly enhanced cell migration capacity (achieving 79.39% wound closure at 48 h), reduced reactive oxygen species (ROS) accumulation, alleviated mitochondrial membrane potential aberrations, and decreased interleukin-17 (IL-17) release by 63%. Concurrently, it markedly elevated key skin barrier protein levels in HaCaT cells, notably enhancing filaggrin (FLG, 1.82-fold) and lamellar oligomerizing protein (LOR, 2.04-fold) expression. Furthermore, PP-FP maintained epidermal thickness, suppressed formation of DNA damage marker γ-H2AX, downregulated matrix metalloproteinase-3/9 (MMP3/9) expression by 40–50%, and effectively preserving epidermal barrier function. This study demonstrates that fermentation-derived *Passiflora edulis* Sims polysaccharides mitigate UVB damage through a dual mechanisms—antioxidant protection and barrier reinforcement—providing a sustainable strategy to repurpose agricultural byproducts for high-efficacy skincare formulations.

**Graphical abstract:**

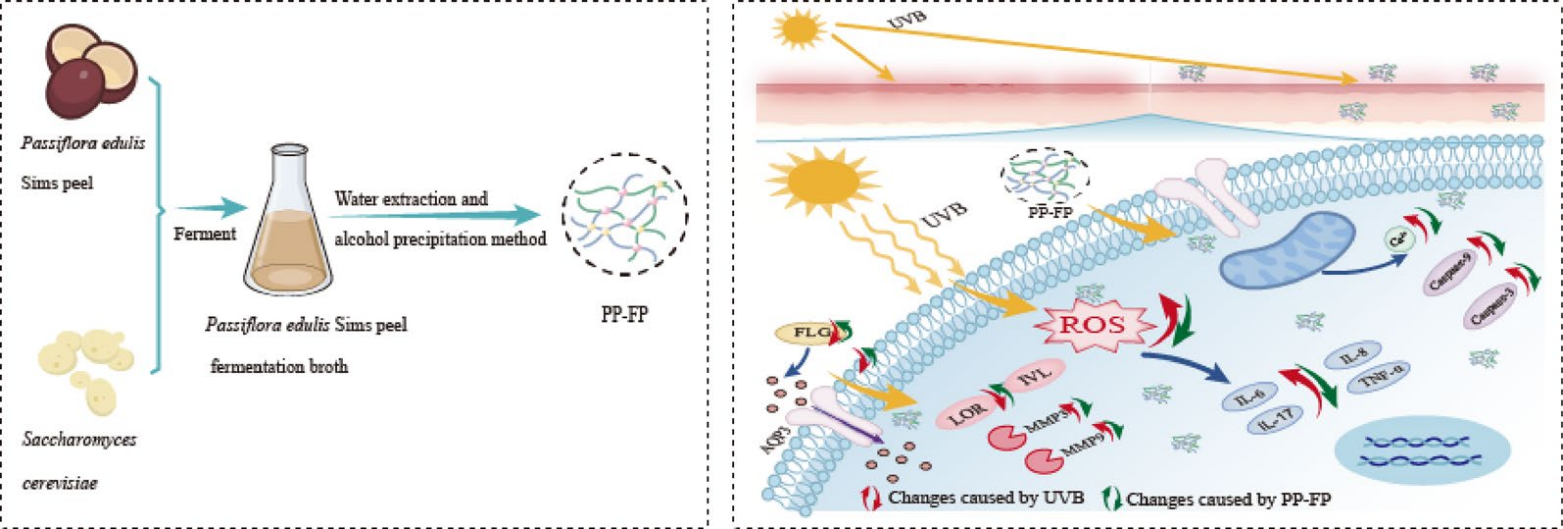

## Introduction

The outer peel of *Passiflora edulis* Sims accounts for 50% of the fruit’s full weight and is rich in natural bioactive compounds, including polysaccharides and polyphenols(Chen et al. 2023a). Polysaccharides in the peel are important bioactive substances. Mung beans peel and Pomelo peel polysaccharides exhibit significant antioxidant properties, which can effectively neutralize free radicals and mitigate cellular damage to delay cellular aging(Lin and Huang 2025; Pardo Solórzano et al. 2024). Additionally, their anti-inflammatory and immunoregulatory effects contribute to reducing cellular inflammation and enhancing immune function(Teng et al. 2022a). As a significant byproduct of the *Passiflora edulis* Sims industry, *Passiflora edulis* Sims peel holds great potential for utilization. However, large quantities of peel are currently discarded as waste(Zhivkova 2023), resulting in resource wastage and environmental concerns. Through technological innovation and industrial chain extension, the utilization of *Passiflora edulis* Sims peel can be enhanced, transforming it into valuable products. This approach promotes resource recycling and supports the sustainable development of the *Passiflora edulis* Sims industry. Novel development strategies and industrial applications for these wastes are urgently needed.

In recent years, research on the skin benefits of *Passiflora edulis* Sims peel is gaining increasing attention, with the protective effects of fermented passion fruit on the skin drawing particular interest. However, despite the field’s significant research potential, studies *Passiflora edulis* Sims peel polysaccharides remain relatively limited. Numerous studies have demonstrated that plant polysaccharides possess anti-inflammatory properties(Flórez-Fernández et al. 2023; Valdés-González et al. 2023; Jen et al. 2021), while fermentation processes can enhance their bioactivity, potentially leading to superior antioxidant and anti-inflammatory effects(Prete et al. 2024; Ai et al. 2020), These properties may help repair and protect the skin from ultraviolet damage(Zhang et al. 2022b). Against the backdrop of resource recycling and green development, transforming *Passiflora edulis* Sims peel—a byproduct of juice processing—into high-value raw materials rich in polysaccharide bioactive compounds holds innovative significance. Through fermentation characterization and active ingredient extraction, these materials can be effectively applied in cosmetic functional development. This strategy not only reduces the waste of biological resources but also alleviates environmental pressure, serving as a critical measure for sustainable development. Further research investigation into the skincare benefits of fermented *Passiflora edulis* Sims peel could not only elucidate its biological mechanisms but also provide new scientific evidence and technical support for developing highly effective natural sunscreen skincare products.

Ultraviolet (UV) radiation is electromagnetic radiation emitted by the sun, classified into three categories based on wavelength: UVA, UVB, and UVC. UVB radiation has a wavelength between 280 and 320 nanometers, accounting for only about 5% of the total UV radiation reaching Earth’s surface. However, due to its high energy, UVB causes significant skin damage(Dusabimana et al. 2024; Kageyama and Waditee-Sirisattha 2019). It directly damages skin cells, triggering acute inflammatory responses. Common symptoms include redness, swelling, pain, and peeling, with blister formation in severe cases. UVB radiation induces cellular DNA damage and promotes excessive production of ROS. Moderate ROS are essential for cellular survival, but excessive accumulation disrupts mitochondrial function and induces cell death, triggering oxidative stress(Mapoung et al. 2021; Panieri and Santoro 2016). This oxidative stress, in turn, triggers acute skin inflammation by stimulating the release of inflammatory mediators such as cytokines and chemokines, which manifest as symptoms as redness, warmth, and pain in the skin(Tang et al. 2017; Zhang et al. 2022a).

There is a strong correlation between the stability of epidermal barrier function and response speed of skin steady-state recovery after sunburn. Disruption and inactivation of barrier-related proteins can compromise the epidermal barrier, leading to significant damage. For example, deficiencies in proteins such as filaggrin (FLG), Caspase 14, loricrin (LOR), and aquaporin-3 (AQP3) contribute to increased water loss, reduced synthesis of moisturizing factors between skin cells(Lee et al. 2017; Hoste et al. 2011), disorganization of the lipid bilayer, decreased cohesion of stratum corneum cells, weakened mechanical integrity, and diminished photoprotective effects(Nemoto-Hasebe et al. 2009). As a result, the skin becomes dry, rough, and susceptible to cracking and peeling, ultimately disrupting skin homeostasis(Lee and Lee 2014; Park et al. 2020). Furthermore, damage to the skin barrier compromises its ability to resist harmful external substances, increasing susceptibility to infections and irritation. Given the inevitability of UV exposure, there is an urgent need to develop more effective strategies to protect the skin and mitigate UV-induced inflammation and barrier damage. Studies have shown that a variety of natural extracts and biomolecules with antioxidant activity can protect skin cells from light damage and abnormal premature aging. This provides a new solution to alleviate the skin photoinflammation caused by ultraviolet radiation(Cruciani et al. 2025).

In this study, we selected *Passiflora edulis* Sims peel for yeast fermentation, employing Saccharomyces cerevisiae. *Passiflora edulis* Sims peel fermented PP-FP was obtained by isolating the fermentation product. The effects on the skin of direct exposure to ultraviolet B radiation and the topical application of PP-FP to repair ultraviolet B damage were compared. This study established a UVB-induced HaCaT cell damage model to investigate the effects of PP-FP on DNA damage, mitochondrial function, ROS levels, cell survival rate, inflammatory response, and skin barrier function in HaCaT cells. Furthermore, an animal model was established to evaluate dorsal skin damage and pathological changes in mice skin using histopathological analysis, thereby further exploring PP-FP’s protective effects against UVB-induced inflammation and skin barrier impairment. We are committed to enhancing the utilization rate of *Passiflora edulis* Sims peel through technological innovation and industrial chain extension, transforming it into high-value-added products to achieve resource recycling and promote the sustainable development of the *Passiflora edulis* Sims industry.

## Materials and methods

### Materials and reagents

*Passiflora edulis* Sims peel was collected from Yunnan, China. Saccharomyces cerevisiae was provided by the Culture Collection Center of the Beijing Institute of Food and Brewing (Beijing, China). HaCaT human immortalized keratinocytes were purchased from the China Institute of Inspection and Quarantine (Beijing, China). Cell culture reagents, including DMEM medium, fetal bovine serum, streptomycin, penicillin, and 0.25% trypsin-EDTA, were from GIBCO Life Technologies (California, USA). CCK-8, DEPC water, First-Strand cDNA Synthesis Kit, Fast Super EvaGreen qPCR Master Mix Kit, and MMP3/MMP9 assay kits were supplied by Biorigin Biotechnology Co., Ltd. (Beijing, China). Total RNA was extracted using TirQuick reagent (Solarbio Life Sciences Technology Co., Beijing, China). Assay kits for ILs, TNF-α, FLG, AQP3, IVL, and LOR were purchased from Wuhan Huamei Bioengineering Co., Ltd. (Wuhan, China), while INV and LOR ELISA kits were from CLOUD-CLONE CORP. (Wuhan, China). ROS detection kit, DAPI nuclear staining solution, PMSF, γ-H2AX immunofluorescence detection kit, JC-1 mitochondrial membrane potential assay kit, and Ca²⁺ fluorescent probe kit were obtained from Beyotime Biotechnology Co., Ltd. (Shanghai, China). BALB/c mice were provided by Beijing Vital River Laboratory Animal Technology Co., Ltd. (Beijing, China). FLG and LOR antibodies were purchased from Beijing Bioss Biotechnology Co. (Beijing, China).

### Sample preparation

The initial preparation involved subjecting pristine, whole *Passiflora edulis* Sims peels to a washing step with deionized water, after which they were dehydrated in an oven at 60 °C for 42 h. The resulting dried peel was comminuted using a mechanical grinder, and the particles were size-fractionated through a 100-mesh sieve. For the subsequent extraction, the powdered substrate was suspended in deionized water at a 1:40 ratio and subjected to autoclave treatment at 121 °C for 15 min. After cooling to room temperature, 5% (v/v) *Saccharomyces cerevisiae* suspension was added. The mixture was stirred at 28 °C and 180 rpm for 48 h to promote yeast growth. It was then centrifuged at 5000 rpm for 10 min. The resulting supernatant was collected and freeze-dried for subsequent use.

A mixture was prepared by combining the fermented *Passiflora edulis* Sims peel extract with pure ethanol at a volumetric ratio of 1:3. Polysaccharide extraction was then carried out through an ethanol-induced precipitation technique. The resulting mixture was refrigerated at 4 °C for overnight sedimentation, and this precipitation step was repeated several times to maximize the polysaccharide recovery rate and reduce co-precipitated impurities. Redissolved in ultrapure water, the precipitated polysaccharides were treated with a 3-fold volume of Sevag reagent to denature and remove residual proteins. Following centrifugation at 5000 rpm for 10 min, the obtained supernatant was subjected to 72 h dialysis (10 kDa MW cutoff), and the dialyzed polysaccharide solution was subsequently freeze-dried to yield the fermented crude polysaccharide from *Passiflora edulis Sims* peel fermentation, designated as PP-FP.

### Structural characterization of PP-FP

#### Atomic force microscope (AFM) and scanning electron microscope (SEM)

The images were analyzed using a Dimension Icon atomic force microscope (AFM), which has a maximum lateral scan size of 90 μm × 90 μm. Following gold plating, morphological features of PP-FP were measured using employing a FEI Nova Nano SEM 450 field emission scanning electron microscope.

After being fixed onto sample stubs with conductive adhesive, the samples were sputter-coated with gold at an accelerating voltage of 5 kV, with scale bars of 100, 50, 20, and 10 μm. An FEI Nova Nano SEM 450 instrument was utilized to characterize the morphology of the polysaccharides.

#### Fourier transform infrared spectroscopy (FT-IR)

The structure of PP-FP was characterized by Fourier transform infrared spectroscopy (FT-IR). The sample powder was mixed with potassium bromide in a 1:100 ratio, thoroughly ground, and pressed into pellets; spectra were then acquired in transmission mode using a Vertex 70 spectrometer. The scanning range was 4000–600 cm⁻¹ with a resolution of 1 cm⁻¹, and the scan was performed 32 times. The absorption peaks were then analyzed in detail.

#### Molecular weight determination by GPC-LS-IR

The sample was dissolved in the mobile phase (0.1 M NaNO₃ containing 0.05% NaN₃) at a concentration of 2 mg/mL and filtered through a 0.22 μm membrane prior to injection. Separation was performed on a column maintained at 35 °C, with a flow rate of 0.6 mL/min. Narrowly distributed pullulan polysaccharides were used as standards, with peak molecular weights including 642, 334, 49.4, 22, and 6.3 kDa. The number-average molecular weight (Mn), weight-average molecular weight (Mw), and polydispersity index (Mw/Mn) were calculated.

#### Monosaccharide composition analysis

Reference materials for the precise measurement of glucose, ribose, rhamnose, glucuronic acid, galacturonic acid, and related monosaccharides. These reference substances were dissolved in water and diluted to prepare a mixed reference solution, with a concentration of 50 µg/mL for each monosaccharide component. Weigh the sample and transfer it to a 10 mL ampoule. Add 3.0 mL of 2 mol/L trifluoroacetic acid, purge the ampoule with nitrogen to displace air, then seal it. Perform acid hydrolysis at 120 °C for 4 h. Following the addition of methanol, the solution was sparged with nitrogen to eliminate trifluoroacetic acid. Subsequently, the residue was reconstituted in 3.0 mL of water. and the final concentration of the sample solution was adjusted to 50 µg/mL.

A working solution was formulated by combining 0.6 M sodium hydroxide, 0.4 M PMP-methanol, and methanol in a volumetric ratio of 1:1:2. The mixture was incubated at 70 °C for one hour to facilitate the reaction. Following a 10-minute cooling period in an ice-water bath, the solution was neutralized with 0.3 M hydrochloric acid. Thereafter, 1 mL of chloroform was introduced, and the sample was agitated vigorously for one minute, then centrifuge at 3000 rpm for 10 min. This liquid-liquid extraction procedure was carried out in triplicate. The derivatization procedure for the sample was performed as described, and the supernatant was collected for HPLC analysis.

The quantification of PP-FP was conducted by high-performance liquid chromatography (HPLC) employing an Xestaral C18 column (4.6 × 200 mm, 5 μm). The mobile phase consisted of a mixture of acetonitrile and an aqueous potassium dihydrogen phosphate buffer. The analysis was performed under the following conditions, flow rate: 1.0 mL/min Column oven temperature: 30 °C Injection volume: 20 µL Detection performed using a UV detector at a wavelength of 250 nm.

### Cell culture and determination of cell viability

According to the reference*(*Zhang et al. 2022c), cell cultures were maintained in Dulbecco’s Modified Eagle Medium (DMEM) supplemented with 10% fetal bovine serum (FBS) and 1% penicillin-streptomycin. Cells were cultured for 48–72 h at 37 °C in a humidified environment with 5% CO₂. When cells reached 80% confluence, they were digested with trypsin for passage and subsequent experiments.

Cell viability of HaCaT cells was determined using the CCK-8 assay. Cells were seeded at a density of 1 × 10^4^ cells/well in 100 µL volumes in a 96-well plate and cultured for 12 h. Control wells and blank wells received 100 µL of serum-free DMEM medium, while sample wells received an equal volume of different sample concentrations. After 24 h, discard the medium. Then add 100 µL serum-free DMEM and 10 µL CCK-8 solution, and continue incubating for 3 h. Following this, determine the absorbance at 450 nm to calculate the percentage of viable cells.

Based on the damaging UVB dose identified in our previous study *(*Jiang et al. 2025), a UVB-induced HaCaT cell injury model was established at a radiation dose of 40 mJ/cm². Cells were seeded into 96-well plates and cultured for 24 h following the aforementioned protocol. After medium removal and PBS washing, sample wells were treated with 100 µL of samples at gradient concentrations, while control, blank and model wells were given an equal volume of serum-free DMEM instead. Following 24 h incubation, cells in the model and sample groups were subjected to UVB radiation, and cell viability was subsequently determined using the aforementioned method.

Cell viability (%) = [(Absorbance of sample well - Absorbance of blank control) / (Absorbance of cell control - Absorbance of blank control)] × 100%.

### Cell scratching assay

Following trypsinization, cells were plated at a density of 6 × 10⁴ cells per well in 6-well plates, each containing 2 mL of DMEM growth medium. To minimize variations in initial cell density between experimental groups, cells were resuspended after digestion and gently pipetted at least 10 times to obtain a homogeneous single-cell suspension. An equal volume of the cell suspension was added to each well, and the plates were gently swirled to ensure even distribution. All experimental groups were seeded simultaneously from the same batch of cell suspension to eliminate batch-to-batch variation. After a 24-hour incubation period to allow for cell adhesion, a 200 µL sterile pipette tip was used to create a continuous, straight and uniformly wide linear scratch vertically in the center of the cell monolayer in each well. Uniform force was maintained throughout the process to avoid inconsistent scratch widths. Then gently wash three times with phosphate-buffered saline (PBS) to remove any detached cells. Subsequent to the application of experimental treatments, all cultures, including both the control and test groups, were subjected to UVB radiation for a 24-hour period. Cell migration into the wound area was monitored, and images were captured at the 24-hour and 48-hour time points. The extent of wound closure was quantified by measuring the remaining cell-free area using ImageJ software.

Cell migration rate (%) = [(Initial scratch area - Scratch area at different time points) / Initial scratch area] × 100%.

### ROS, calcium Influx and mitochondrial membrane potential

Establish a control group, model group, and sample group. Seed HaCaT cells (5 × 10⁵ cells) into 6-well plates and culture for 24 h. Cells in the control and model cohorts were supplemented with serum-free MEM, while the experimental groups were treated with PP-FP at doses of 125, 250, and 500 µg/mL. After 24 h, the model and experimental cohorts were subjected to an 80-second UVB exposure. A subsequent 12-hour incubation at 37 °C preceded the following assays.

Intracellular ROS were quantified with the DCFH-DA fluorogenic dye. The cell cultures were incubated in a 1:1000 dilution of serum-deprived medium for 20 min at 37 °C. To assess cytosolic calcium entry, the Fluo-4 AM ester dye was applied at a concentration of 4 µM. A volume of 1 mL of the dye solution was introduced to each well, followed by a 30-minute incubation period at 37 °C. The mitochondrial membrane potential was assessed using the JC-1 potential probe. A 50 µL aliquot of the 200× concentrated solution was mixed with 8 mL of ultrapure water, followed by the addition of 2.0 mL of 5× JC-1 staining buffer to prepare the JC-1 working solution. Subsequently, 1 mL of the prepared working solution was dispensed into each well, and the plate was incubated at 37 °C for 20 min. Detection of ROS, calcium influx, and mitochondrial membrane potential strictly followed the protocols provided by the respective reagent kit manufacturers.

### Enzyme-linked immunosorbent assay (ELISA)

The HaCaT cells (5 × 10⁵ cells per well) were seeded into a 6-well plate and processed as described above. After collecting the supernatant, cells were lysed by adding PMSF at a 1:100 ratio and cell lysis buffer, with 200 µL of cell lysis buffer added to each well. The lysate was then cultured with antibodies or antigens immobilized on the kits. Standard samples were diluted, and then both the sample and standard solutions were added to the ELISA plate. After incubation, the plate was washed, and the chromogenic substrate and stop solution were sequentially added. The detailed experimental procedures were performed according to the instructions provided with the inflammatory-related factor, skin barrier-related factor, and other detection kits.

### Animals

#### Experimental Design and UVB Irradiation

The ethical oversight for all in vivo work in this investigation was provided by the Scientific Research Ethics Committee of Beijing Technology and Business University (Authorization Code: 2025–192). After 7 days of adaptive feeding, the BALB/c mice were randomly divided into 5 groups with 6 individuals in each group. The groups were designated as follows: control group, model group, LG-m (low-dose sample group, 60 mg/mL PP-FP), MG-m (medium-dose sample group, 120 mg/mL PP-FP), and HG-m (high-dose sample group, 240 mg/mL PP-FP).

Each day, 250 µL of PP-FP solution (or normal saline for control and model groups) was evenly applied to the shaved dorsal skin of the mice. Thirty minutes after application, the dorsal skin of mice in the model and treatment groups was exposed to UVB irradiation at a dose of 300 mJ/cm². This procedure was repeated once daily for seven consecutive days. On the eighth day, mice were euthanized by cervical dislocation. Dorsal skin tissues were collected, divided into portions for histology, RNA extraction, and protein analysis, and stored at − 80 °C or fixed in 4% paraformaldehyde.

#### Histopathology

Skin specimens were fixed in a 10% formaldehyde solution and subsequently processed for paraffin embedding. Tissue sections were cut to a 6-micrometer thickness and dyed employing the hematoxylin-eosin (H&E) technique. The staining protocol was performed as follows: sections were immersed in hematoxylin for 3 min to stain nuclei, then rinsed thoroughly with running water to remove excess dye. Sections were then dehydrated with gradient ethanol: 70% ethanol for 10 min and 90% ethanol for 10 min, to remove tissue moisture stepwise. After dehydration, sections were counterstained in 0.5% eosin solution for 2–3 min to stain cytoplasm and extracellular matrix, boosting tissue contrast. Finally, sections were dehydrated in anhydrous ethanol, cleared with xylene, and mounted with cover slips for subsequent pathological observation.

#### Immunofluorescence

Tissue sections were heat-fixed at 60℃ for 1 h to secure slices to slides and maintain tissue integrity during subsequent processing. Deparaffinization and rehydration were performed via sequential xylene and graded ethanol washes to remove paraffin and restore tissue moisture. Samples were immersed in fixative solution for 10 min to stabilize antigens, followed by thorough PBS rinsing to eliminate residual fixative. .Endogenous peroxidase activity was blocked by applying a blocking solution for 15 min at ambient temperature. Sections were incubated with a specific rabbit monoclonal antibody against γ-H2AX at room temperature for 1 h to enable specific antigen-antibody binding. After three PBS washes to remove unbound primary antibody, the secondary antibody was added and incubated at room temperature for another 1 h to amplify the signal. After two additional washes, DAPI nuclear staining solution was added and cultured in the dark for 5 min. Sections were rinsed with PBS to remove unbound DAPI, mounted with glycerol mounting medium, and visualized under a microscope at 400× and 100× magnification.

#### Quantitative real-time RT-PCR (qRT-PCR)

The tissue was retrieved from the − 80 °C freezer and cut into small fragments. Transfer the fragmented tissue into a homogenizer, add 1 mL of Trizol reagent, and homogenize the mixture until it is fully emulsified. Maintain the sample at ambient temperature for 5 min. Subsequently, pellet the material by spinning at 12,000 g for 5 min in a refrigerated (4 °C) centrifuge. Carefully collect the resulting supernatant into a new tube certified to be nuclease-free and introduce 200 µL of chloroform. Agitate the solution vigorously and let it stand at room temperature for a further 5 min. Repeat the centrifugation step under the same conditions (4 °C, 12,000 g) for a duration of 15 min. Then, carefully aspirate the upper aqueous phase and transfer it to a new nuclease-free tube for nucleic acid collection. Add an equal volume of isopropanol to the aqueous phase, and let the mixture stand at room temperature for 10 min to facilitate nucleic acid precipitation. Centrifuge at 4 °C and 12,000 g for 15 min to pellet the RNA. After discarding the liquid supernatant, a minor whitish precipitate should be visible at the tube’s base. Wash the RNA pellet with 1 mL of pre-cooled 75% ethanol solution, and vortex thoroughly to mix. Centrifuge again at 12,000 g and 4 °C for 10 min, then collect the RNA pellet. Discard the supernatant and remove residual ethanol completely. Air-dry the pellet at room temperature for approximately 10 min. Finally, resuspend the purified RNA in 30 µL of nuclease-free water.

Using the Fast-Strand cDNA Synthesis Kit and Fast Super EvaGreen^®^ qPCR Premix Kit, cDNA was synthesized from extracted RNA and amplified via quantitative PCR using a three-step method. The specific experimental procedure follows Sun et al. The primer sequences are listed in Table 1.

**Table 1:** Primers

Primers	Direction	Primer sequence(5’→3’)
MMP3	F	CCCTGCAACCGTGAAGAAGA
	R	GACAGCATCCACCCTTGAGT
MMP9	F	CAGCCGACTTTTGTGGTCTTC
	R	ATAGCGGTACAAGTATGCCTCTG
TNF-α	F	CCCTCACACTCACAAACCAC
	R	ATAGCAAATCGGCTGACGGT
IL-6	F	TGATGGATGCTACCAAACTGGA
	R	TGTGACTCCAGCTTATCTCTTGG
COX-2	F	TTTGCATTCTTTGCCCAGCA
	R	CCTCTCCACCAATGACCTGAT
IL-17 A	F	GAGAGCTTCATCTGTGTCTCTG
	R	GCGCCAAGGGAGTTAAAGAC
ACT1	F	GGCTTCCAAACTGCGATTGA
	R	TGTAAGCCATGCTCGTCCTC
TRAF6	F	ATCCTCATCAGAGAACAGATGCC
	R	GTGTCGTGCCAAGTGATTCC
β-action	F	GCTCCGGCATGTGCAAAG
	R	TTCCCACCATCACACCCTGG

F: forward primer; R: reverse primer.

### Statistical analysis

The data obtained from the experiments were processed with SPSS. (Version 17.0, IBM Inc., Armonk, New York, USA). All measurements are reported as the mean ± standard deviation. GraphPad Prism 8 was used for graphical analysis. Cell migration rates were calculated using Image J software to process the cell scratch images. The statistical significance of differences between the groups was evaluated using a t-test, with a p-value below 0.05 indicating significance.

## Results

### Structural characterization of PP-FP

**Fig. 1:**
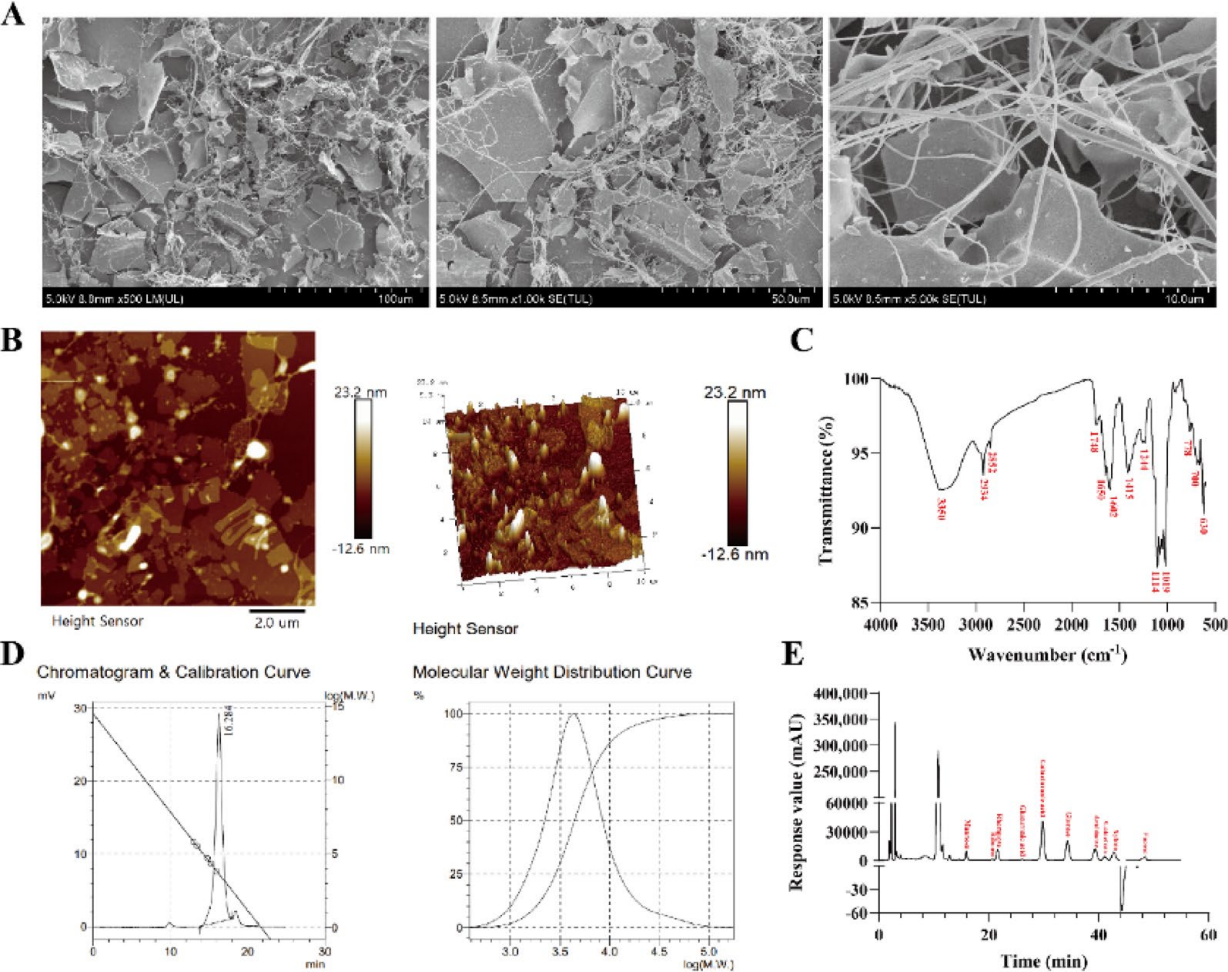
Structural characterization of PP-FP. **A**: SEM images of PP-FP under different magnifications; **B**: AFM image of PP-FP; **C**: FT-IR spectrum of PP-FP; **D**: Molecular weight of PP-FP; **E**: Monosaccharide composition of PP-FP)

AFM and SEM are two complementary and powerful tools for material characterization, which reveal surface and structural information from different dimensions. The structure and micro-morphology of *Passiflora edulis* Sims fermentation polysaccharide (PP-FP) were analyzed using Scanning Electron Microscopy (SEM) and Atomic Force Microscopy (AFM), as shown in Fig. 1A and B. Figure 1A presents the surface morphology of PP-FP at different magnifications. At lower magnification, numerous fibrous and porous structures were observed attached to a flake-like structure, which appeared loose and irregular. At higher magnification, the fibrous structures of PP-FP were intertwined with a relatively uniform width. The loose and porous morphology likely results from CO₂ release and enzymatic degradation during fermentation, which created interconnected pores. The high roughness of the loose structure greatly increases the specific surface area, resulting in a better adsorption capacity of the sample. Figure 1B depicts the surface micro-morphology of PP-FP in a 2D image alongside its corresponding 3D topographical map. The micro-surface morphology appeared as irregular flake-like, while the 3D representation revealed uneven surfaces characterized by sharp peaks and multiple mound-like protrusions, reaching a height of 23.2 nm. The formation of bright particles and valley stack-like structures after fermentation can increase the surface roughness of the samples, potentially enhancing interfacial bonding.

The infrared spectrum of PP-FP shown in Fig. 1C exhibits a broad peak at 3350 cm⁻¹ corresponding to O-H stretching vibrations, indicating the presence of intermolecular and intramolecular hydrogen bonds within the polysaccharide. Spectral bands within the 3000–2800 cm⁻¹ range are indicative of carbohydrates. The signals observed at 2934 cm⁻¹ and 2852 cm⁻¹ are ascribed to C-H stretching modes within the polysaccharide structure. The band at 1748 cm⁻¹ is consistent with the stretching mode of carbonyl (C = O) bonds. The signal at 1608 cm⁻¹ is associated with both the presence of water of crystallization in the polysaccharide and the stretching of C = O bonds in acetyl moieties (NHCOCH₃). Spectral features in the 1400–1200 cm⁻¹ region arise from C-H bending vibrations, whereas those in the 1200–1000 cm⁻¹ range are attributed to furanose ring deformations and C-O stretching modes. More specifically, the bands at 1114 cm⁻¹ and 1019 cm⁻¹ are likely derived from the stretching vibrations of the C-O-C and C-O-H linkages in the pyranose ring, suggesting the existence of pyranosyl glycosidic structures in PP-FP. As sugars like galactose and glucose are predominantly found in their pyranose configuration. This conclusion is consistent with the results of the monosaccharide composition analysis.

**Table 2:** Determination of PP-FP molecular weight

Name	Mn (Da)	Mw (Da)	Mz(Da)	Mw/Mn	Mz/Mn
PP-FP	3.519 × 10^3^	6.66 × 10^3^	1.7304 × 10^4^	1.8925	2.5982

**Table 3:** Monosaccharide composition of PP-FP

Monosaccharide ratio (%)	Area (mAU·min)	Height (mAU)	Area(%)
mannose	215,823	10,298	4.928
ribose	26,653	956	0.609
rhamnose	299,422	10,964	6.836
glucuronic acid	41,706	1115	0.952
galacturonic acid	1,495,395	40,374	34.143
glucose	889,031	21,168	20.298
galactose	569,983	12,068	13.014
xylose	203,201	4195	4.639
arabinose	451,650	8795	10.312
Total	4,379,825	1140	100

The molecular weight and monosaccharide composition of *Passiflora edulis* Sims peel polysaccharide is analyzed as shown in Fig. 1D and E, and Tables 2 and 3. The molecular weight of PP-FP was determined using GPC-LS-IR, revealing a single peak, indicating that PP-FP is a relatively pure monosaccharide. Analysis of the PP-FP polymer revealed a weight-average molecular weight of 6660 Da and a number-average molecular weight of 3519 Da. Its sugar components primarily consist of galacturonic acid, glucose, galactose, arabinose, and rhamnose. The monosaccharide components—galacturonic acid, glucose, galactose, arabinose, and rhamnose—collectively confer moisturizing properties and prebiotic effects to the product due to their hygroscopic characteristics and prebiotic potential. Galacturonic acid (a major component of pectin) and arabinose enhance water retention by forming hydrogels, while glucose and galactose support microbial growth as energy sources for beneficial gut bacteria. Rhamnose, though less common, may modulate bacterial adhesion and short-chain fatty acid production. Together, these sugars synergistically improve skin or mucosal hydration while fostering a balanced microbiome, as demonstrated in polysaccharide fermentation studies. This suggests that *Passiflora edulis* Sims fermented polysaccharides possess good moisturizing and micro-ecological regulation functions.

### PP-FP enhances cell viability and migration after UVB exposure

**Fig. 2:**
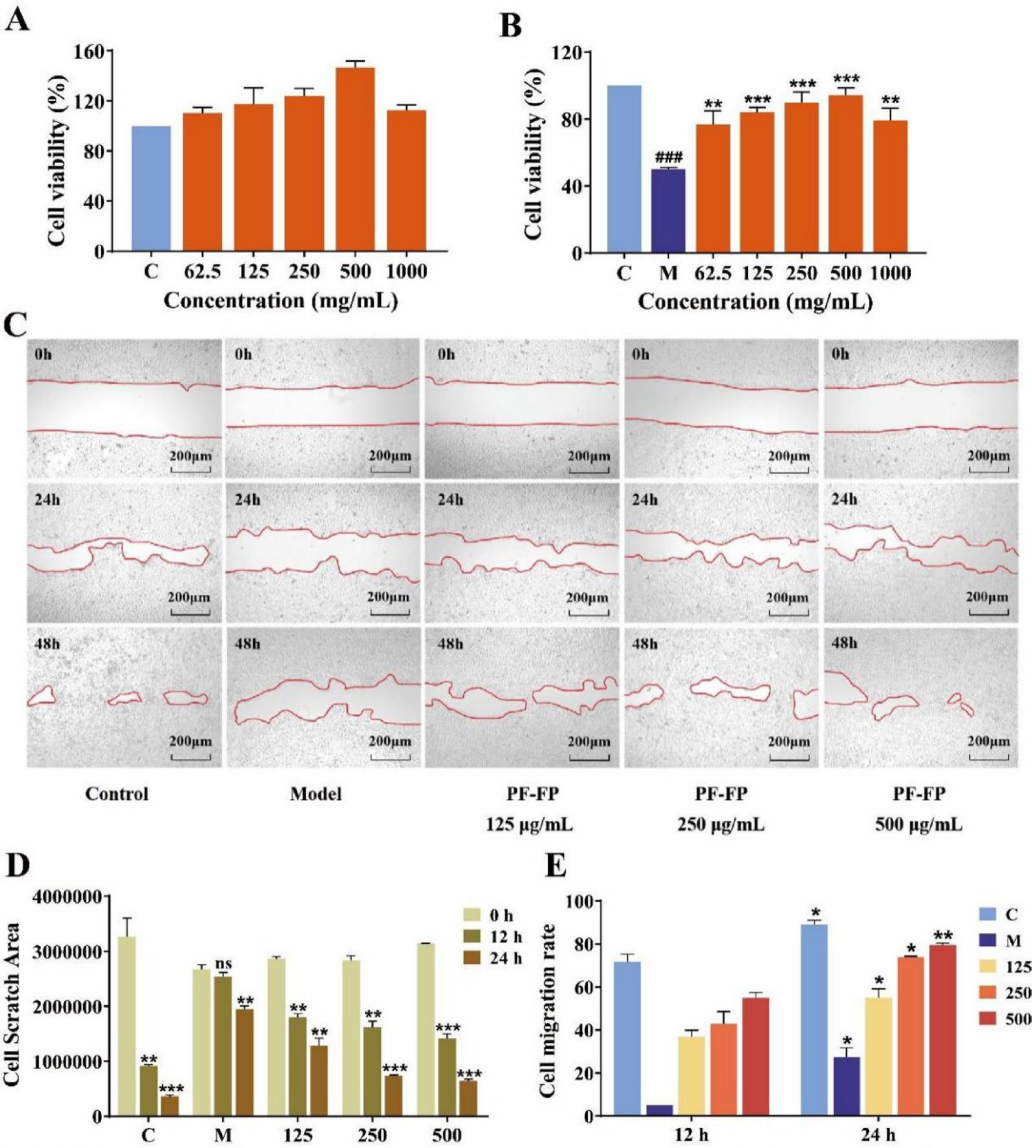
PP-FP Enhances HaCaT cell viability and migration ability. **A** Effect of different concentrations of PP-FP on cell viability; **B**: Protective effect of different concentrations of PP-FP on UVB-damaged cells; **C**: Effect of PP-FP on cell migration ability; **D**: Scratch area of cells at different time points; **E**: Cell migration rate at 12 h and 24 h)

The effects of varying PP-FP concentrations on cellular survival was assessed via the CCK-8 assay (Fig. 2). The data demonstrate that at doses ranging from 62.5 µg/mL to 1000 µg/mL, cell viability was consistently maintained above 80% and was higher than that observed in the control. This suggests PP-FP exhibits no toxic side effects on cells and may promote HaCaT cell proliferation. Cell viability peaked at 500 µg/mL, while proliferation effects diminished at 1000 µg/mL, suggesting a potential stimulatory effect at this concentration. To establish a UVB-induced cellular damage model, HaCaT cells were exposed to a UVB radiation dose of 40 mJ/cm² (as shown in Fig. 2B). In model *group*, exposure to UVB radiation significantly decreased cell viability to 50.19%. However, cells pretreated with PP-FP exhibited markedly higher viability than the model group. Significant protective effects were observed at concentrations of 125 µg/mL, 250 µg/mL, and 500 µg/mL, with cell viability exceeding 80%. A decline in cellular viability was noted at a dosage of 1000 µg/mL, likely resulting from the cytotoxic stress induced by high levels of PP-FP. In subsequent in vitro investigations, these three concentrations were employed as experimental benchmarks, designated as the low- (LG, 125 µg/mL), medium- (MG, 250 µg/mL), and high-concentration (HG, 500 µg/mL) cohorts.

The cell scratch assay simulates the process of epidermal cell migration, allowing for the assessment of cell migration and wound healing capabilities, serving as an in vitro wound healing model(Grimmig et al. 2019). To assess the impact of PP-FP on wound closure, we evaluated its effects on cell migration; these results are displayed in (Fig. 2C, D, E). In the control group, which was not subjected to UVB damage, the cell migration rate reached 88.92% at 48 h, indicating good inherent healing capacity. In the model group, cells exposed to UVB exhibited no significant change in scratch area at 24 h, with a migration rate of only 5.05%, demonstrating poor healing. At 48 h, the migration rate increased slightly to 27.31%, which is less than one-third of the healing seen in the control group cells. As shown in Fig. 2D, cells treated with PP-FP showed a significant reduction in scratch area at 48 h compared to 24 h, indicating their excellent healing capacity. Figure 2E demonstrates that cell migration rate increases with rising PP-FP concentrations. At a concentration of 500 µg/mL, PP-FP treatment resulted in a cell migration rate of 79.39% after 48 h. This value is 2.9-fold greater than that observed in the model group and corresponds to 0.89 times the rate of the control group at the same time point.

The results indicate that UVB exposure leads to a decrease in skin cell migration ability. However, PP-FP mitigates the negative impact of UVB on cellular repair capacity and enhances the migratory ability of HaCaT cells, with the HG group showing the most significant effect.

### PP-FP attenuates oxidative stress and mitochondrial dysfunction

**Fig. 3:**
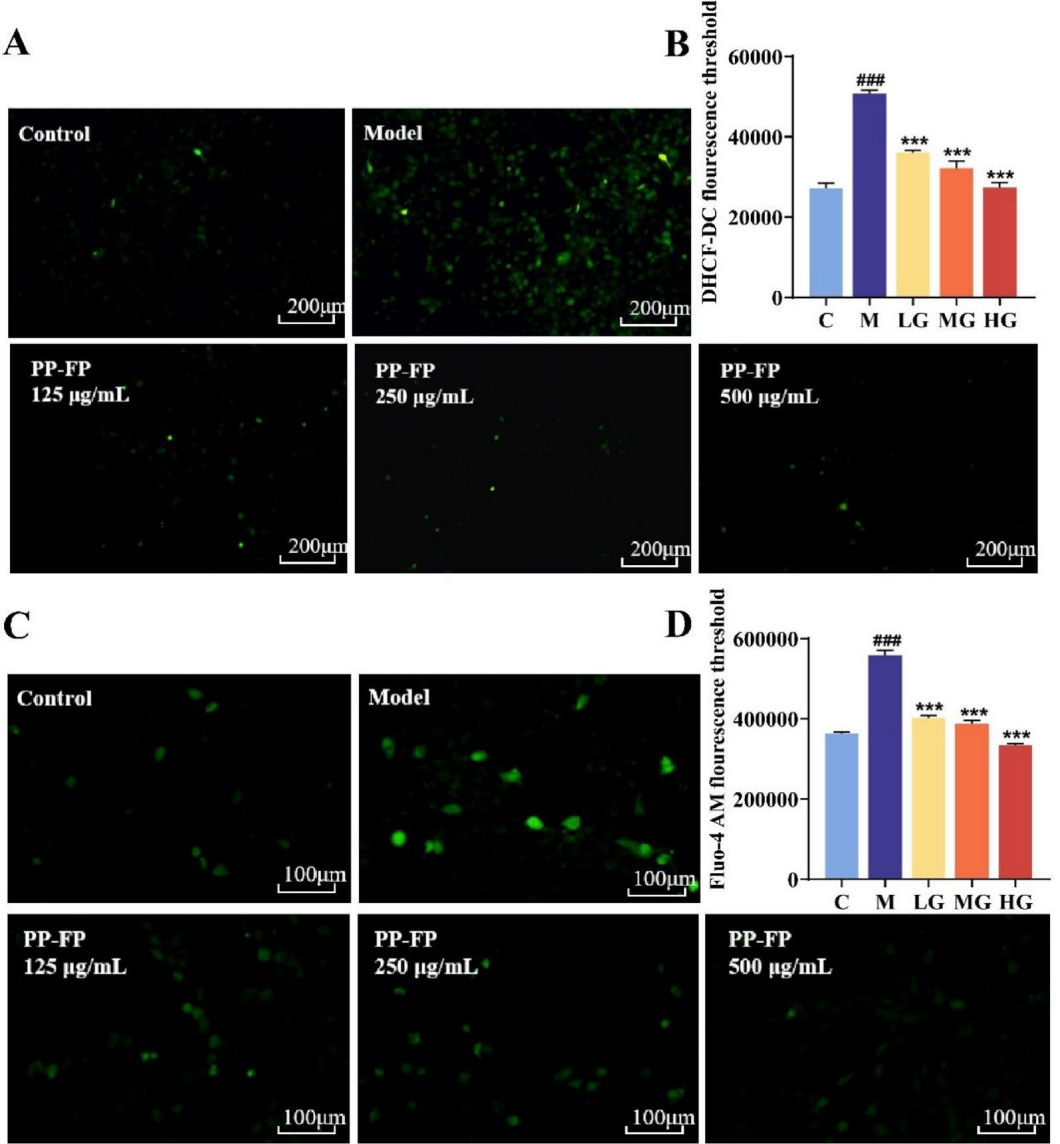
Effects of Different Concentrations of PP-FP on ROS and Calcium Ions in Cells. (**A** ROS fluorescence images; **B**: Fluorescence intensity of ROS;** C**: Calcium ion fluorescence images; **D**: Fluorescence intensity of calcium ions; ### *p* < 0.001, compared with the C group; *** *p* < 0.001, compared with the M group)

The fluorescence intensity of ROS and calcium ions within cells were detected using DHCF-DC and Fluo-4 AM fluorescent probes, respectively. The experimental results are shown in Fig. 3. Following UVB irradiation, the number of green fluorescence signals in HaCaT cells significantly increased, with markedly enhanced fluorescence intensity. Specifically, the fluorescence intensities of DHCF-DC and Fluo-4 AM were 1.86 times and 1.54 times higher than the control group, respectively. After treatment with 125 µg/mL low-concentration PP-FP, the fluorescence intensity significantly decreased. After treatment with 500 µg/mL high-concentration PP-FP, the fluorescence intensity of DHCF-DC and Fluo-4 AM was close to that of the control group. This result shows that PP-FP has the ability to inhibit the accumulation of ROS and calcium ions in cells, alleviating cell inflammation, and normalizing calcium ion influx.

**Fig. 4:**
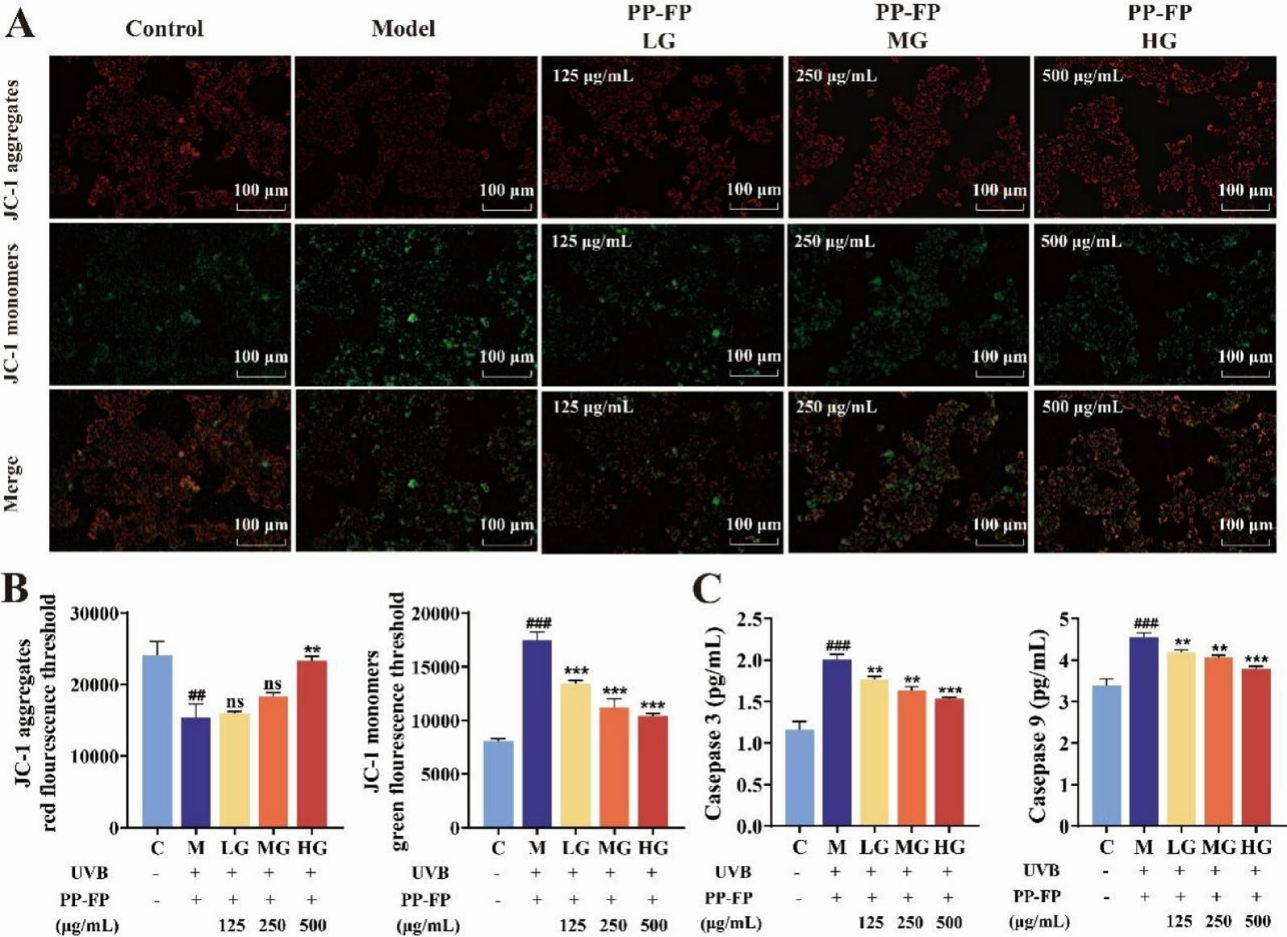
Evaluation of PP-FD Dose-Response on Mitochondrial Health and Apoptosis Induction in Human Keratinocyte (HaCaT) Cultures.** (A** Representative fluorescence images showing changes in mitochondrial membrane potential; **B**: Quantified fluorescence signal of JC-1 aggregates and monomers, indicative of ΔΨm; **C**: Assessment of the activation state of the intrinsic apoptotic pathway via Caspase-3 and Caspase-9 expression.); ## *p* < 0.01, ### *p* < 0.001 compared with group C; ** *p* < 0.01, ***p < 0.001 compared with group M)

One of the earliest events in the apoptotic cascade is the decline in mitochondrial membrane potential, which normal cells maintain at relatively stable levels. Additionally, the structurally similar cysteine proteases—caspases—are distributed throughout the cytoplasm and play a crucial role in mediating apoptosis. Among these, Caspase 3 and Caspase 9 are closely associated with mitochondrias(Mischo et al. 2005). Figure 4 demonstrates the influence of PP-FP on the mitochondrial transmembrane potential and the concentrations of the key apoptosis-associated proteins, Caspase 3 and Caspase 9, in human keratinocyte (HaCaT) cells following ultraviolet B-induced injury.

Changes in fluorescent color can reflect alterations in mitochondrial membrane potential(Jin et al. 2024). In normal mitochondria, JC-1 emits strong red fluorescence as it aggregates into polymers within the mitochondrial matrix. When mitochondrial membrane potential increased, JC-1 exists solely in its free form within the cytoplasm, and resulting in green fluorescence. In healthy cells, a small amount of green fluorescence can be observed, with the majority of fluorescence being red.

As illustrated in (Fig. 4A, B), a marked reduction in red fluorescence intensity was observed in the model group relative to the controls. The fluorescence intensity of the JC-1 aggregates fell to 0.63-fold of the control value. Conversely, the green fluorescence intensity significantly increased, with the JC-1 monomer fluorescence intensity rising to 2.17 times that of the control group. Only a small amount of red fluorescence was observed, with the fluorescence mainly appearing green. After treatment with PP-FP samples, the fluorescence images show a significant increase in red fluorescence, with the JC-1 polymer fluorescence intensity enhanced. The green fluorescence intensity notably decreased, with the JC-1 monomer fluorescence intensity weakened, and only a small amount of green fluorescence was observed, with red fluorescence being dominant. These findings suggest that PP-FP can protect cells from UVB-induced mitochondrial membrane potential reduction, with a significant effect observed at 500 µg/mL PP-FP.

As shown in Fig. 4C, following UVB-induced damage, intracellular caspase levels significantly increased, with Caspase-3 levels nearly doubling compared to the control group. Both caspase-3 and caspase-9 levels were significantly reduced in the high-concentration PP-FP treatment group.

The findings demonstrate that exposure to UVB radiation leads to a reduction in mitochondrial membrane potential and triggers programmed cell death in HaCaT keratinocytes, while PP-FP mitigates apoptosis by stabilizing mitochondrial membrane potential and suppressing intracellular caspase content.

### PP-FP suppresses UVB-induced inflammatory responses

**Fig. 5:**
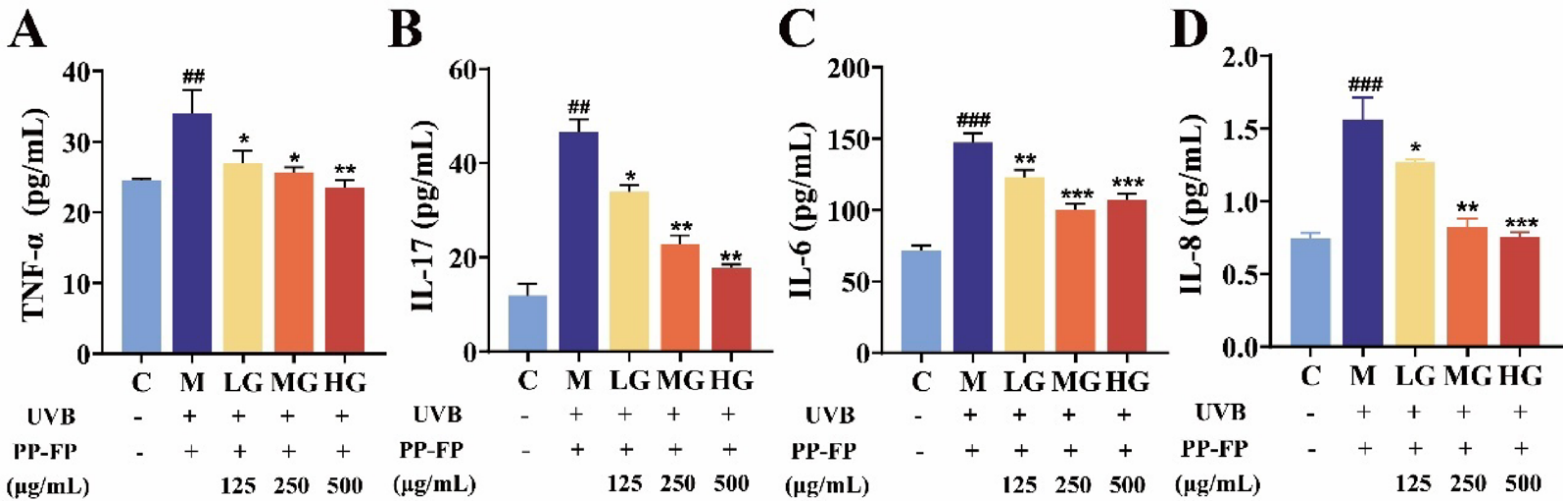
The Effect of Different Concentrations of PP-FP on Inflammatory Factors in HaCaT Cells. (**A**: TNF-α; **B**: IL-17; C: IL-6; D: IL-8; ## *p* < 0.01, ### *p* < 0.001, compared with Group **C** * *p* < 0.05, ** *p* < 0.01, *** *p* < 0.001, compared with Group M)

UVB is the main damaging wavelength of ultraviolet radiation that induces inflammation in epidermal cells(Kim et al. 2023). The effect of PP-FP on pro-inflammatory factor levels in cells is shown in Fig. 5. After UVB exposure, intracellular levels of TNF-α, IL-17, IL-6, and IL-8 significantly increased, confirming that UVB exposure triggers an inflammatory response in skin cells, which in turn leads to damage at other cellular levels.

Administration of PP-FP markedly reduced cellular concentrations of the four pro-inflammatory cytokines. With the exception of IL-6, the suppression of the remaining factors demonstrated a dose-dependent relationship. Furthermore, the MG group demonstrated that PP-FP exerted a stronger inhibitory effect on IL-6, while the HG group reduced intracellular TNF-α and IL-8 levels to undamaged states. These findings confirm that PP-FP significantly suppresses the excessive synthesis of intracellular inflammatory chemokines while effectively alleviating UV-induced cellular inflammatory responses.

### PP-FP restores UVB-disrupted skin barrier function

**Fig. 6:**
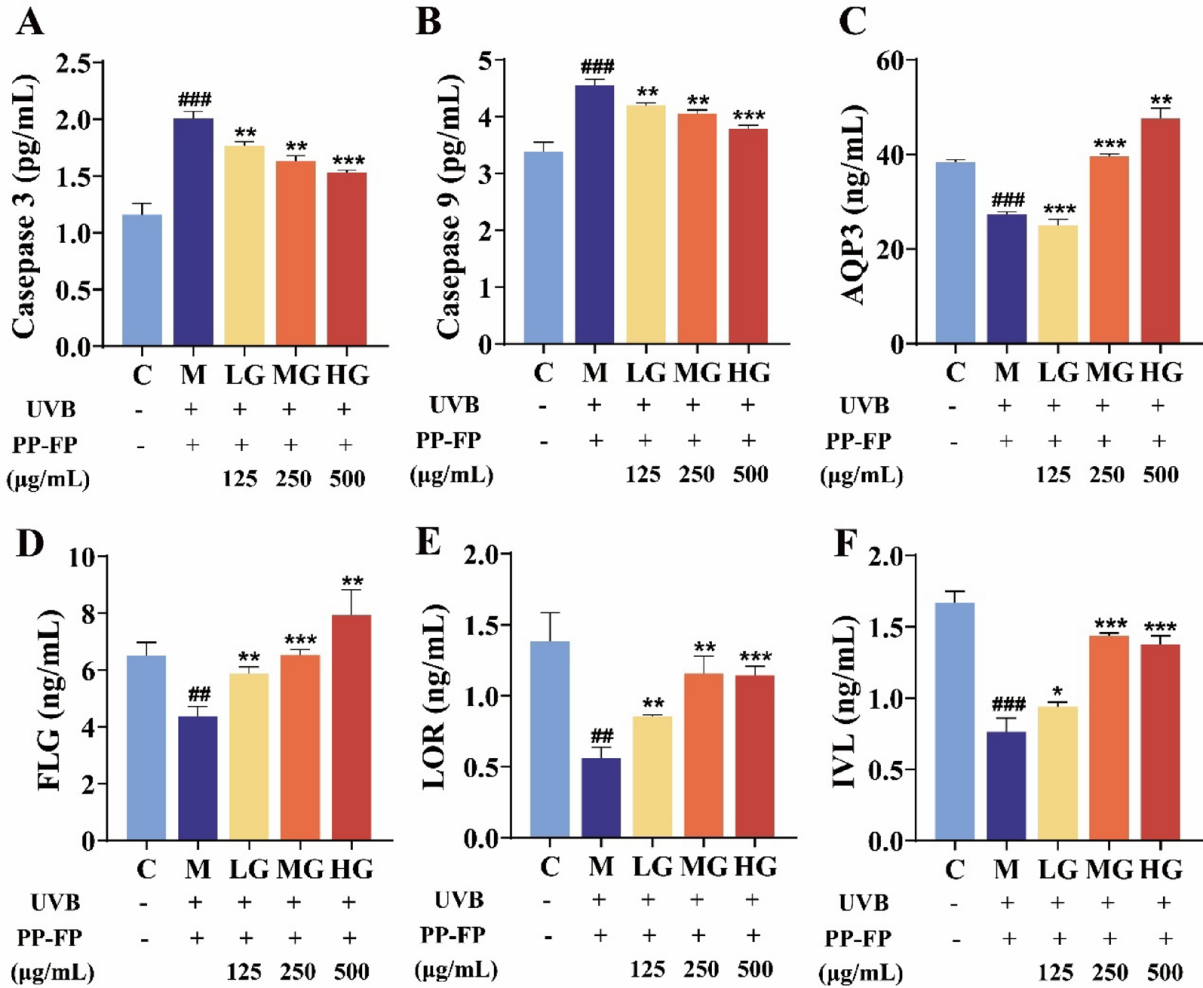
Effects of different concentrations of PP-FP on the levels of skin barrier-related proteins and enzyme activity in HaCaT cells. (**A** MMP3; **B**: MMP9;** C**: AQP3; **D**: FLG; **E**: LOR; **F**: IVL; ## *p* < 0.01, ### *p* < 0.001, compared with the C group; * *p* < 0.05, ** *p* < 0.01, *** *p* < 0.001, compared with the M group)

Figure 6 demonstrates the effect of PP-FP on barrier integrity markers in HaCaT cells. UVB irradiation resulted in a significant upregulation of MMP-3 and MMP-9 activity and a downregulation of AQP3 content compared to the control. As shown in Fig. 6C-D, after UVB irradiation, the expression levels of AQP3, FLG, IVL, and LOR in the cells decreased to varying degrees, this implies that UVB exposure compromises the skin barrier by enhancing protein breakdown and disturbing water homeostasis.

Following treatment with different concentrations of PP-FP, intracellular levels of MMP3 and MMP9 decreased, while those of AQP3, FLG, IVL, and LOR increased to varying degrees. Although no single indicator showed a dose-dependent response, the overall trend indicated that the HG group of PP-FP had the most significant effect. It’s suggesting that PP-FP could mitigate excessive depletion of barrier function proteins, structural instability in cells and a loss of epidermal cell defense ability caused by UVB.

### PP-FP alleviates UVB-induced photodamage in mice

#### Ameliorates epidermal thickening and tissue damage

**Fig. 7:**
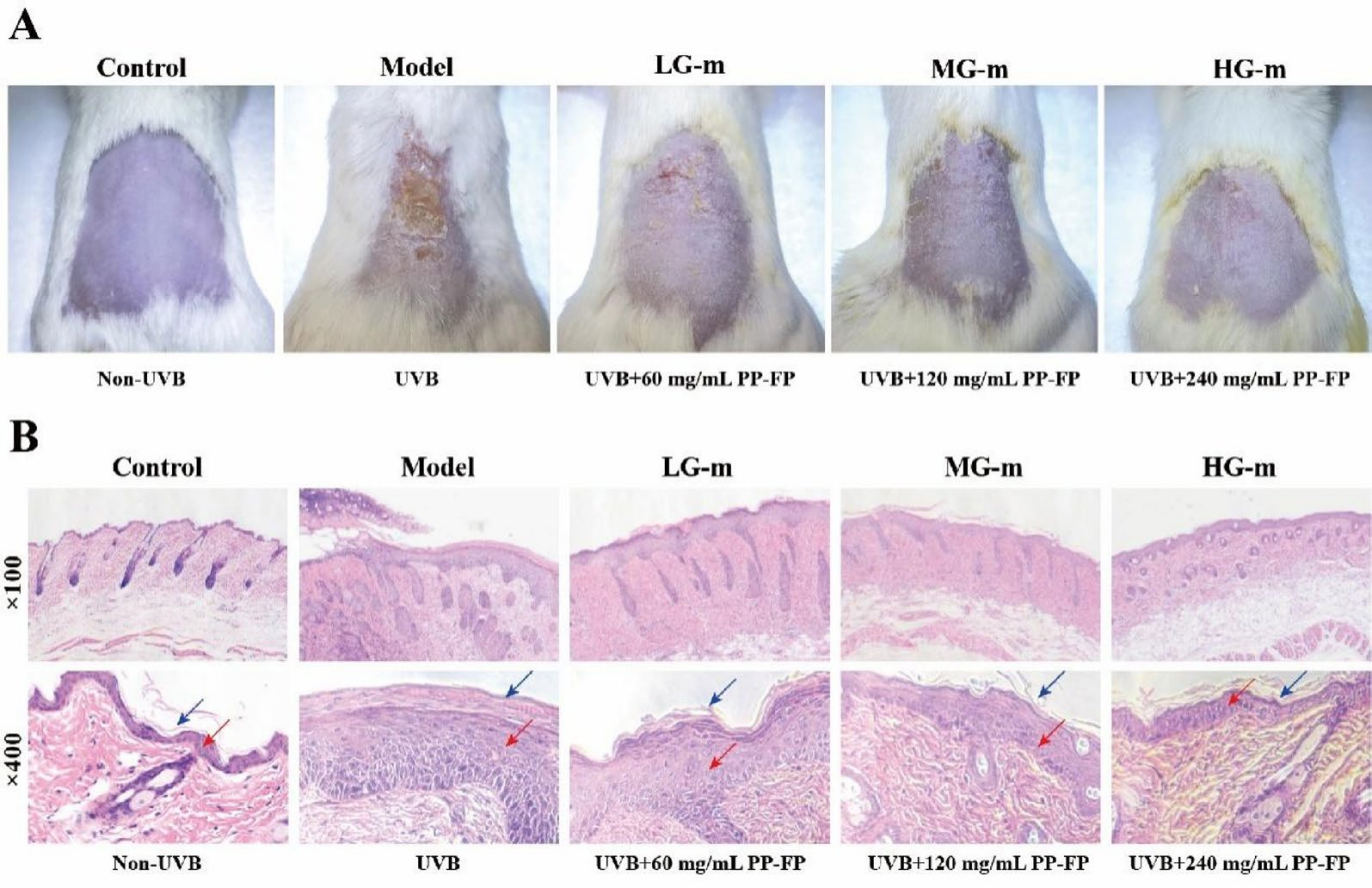
Morphological and histopathological analysis of mouse epidermis

A model of UVB-induced skin damage was established on the dorsal skin of mice using an irradiation dose of 300 mJ/cm² for 7 consecutive days. The skin condition of the mice is shown in Fig. 7. The results show that the model group exhibited dark red, rough skin with severe wounds and thickened scales. After treatment with different concentrations of PP-FP on the mice’s backs, the condition improved. In the LG-m group, the dark red color was reduced, and the wound area decreased significantly. In the MG-m group, no wounds were present on the back, and the scales were notably reduced. In the HG-m group, there were no wounds, only a few scales, and the skin was smooth.

To further investigate the protective effect of PP-FP on mice skin, histopathological changes in skin tissue were analyzed using hematoxylin and eosin (HE) staining. As shown in Fig. 7, relative to the normal controls, animals in the model group demonstrated a marked increase in epidermal thickness (red arrow) along with aberrant keratinization. The stratum corneum appeared denser, with a marked increase in thickness (blue arrow) and hypertrophy of the granular and spinous layers, indicating skin barrier damage caused by UVB irradiation. The experimental results indicate that high concentrations of PP-FP provide superior protective effects on the skin of mice. After treatment with 60 mg/mL of low-concentration PP-FP, the thickening of stratum corneum cells was significantly reduced, and epidermal proliferation was slightly diminished. In the 240 mg/mL PP-FP group, epidermal proliferationand keratinization were significantly reduced, demonstrating strong resistance to UVB-induced damage.

#### Reduces DNA damage and inflammatory gene expression

**Fig. 8 γ-H2AX immunofluorescence staining of mouse epidermal tissue.**
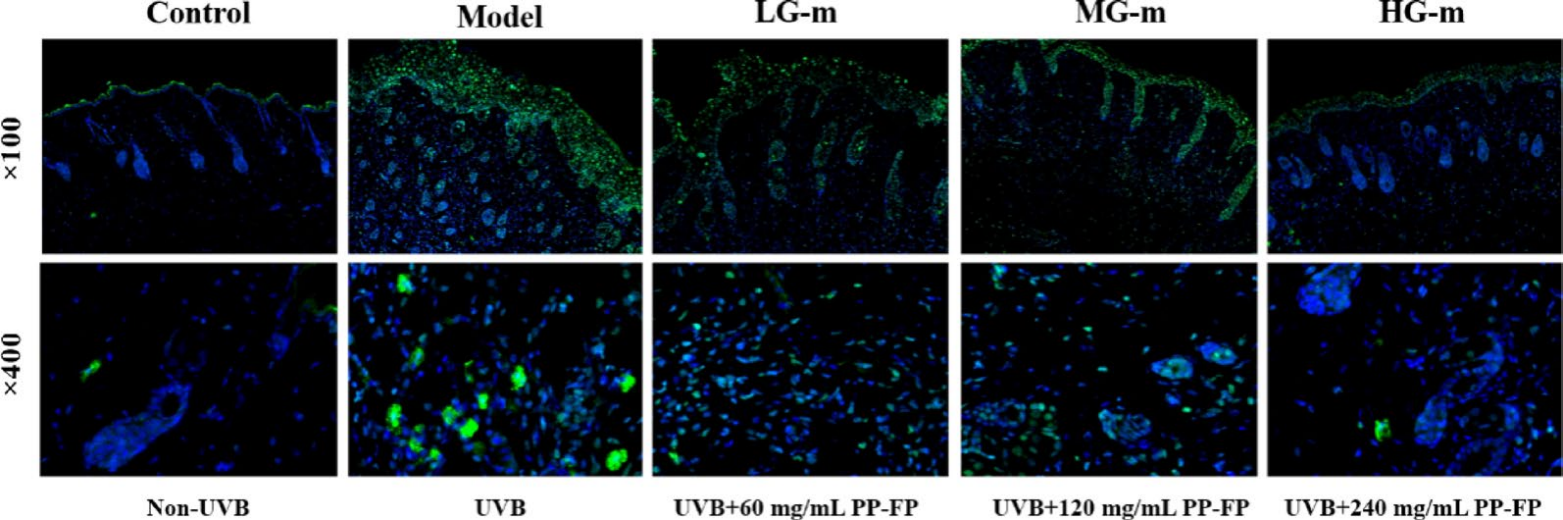
The DNA damage in mice skin tissue was assessed using γ-H2AX immunofluorescence staining, with images captured at different magnifications, as shown in Fig. 8. In the control group, only the stratum corneum exhibited low levels of γ-H2AX, and there was almost no green fluorescence in the nuclei. In the model group, a large accumulation of γ-H2AX was observed in the epidermal stratum corneum and granular layers, with a significant increase in green fluorescence. DNA damage was also observed in the nuclei of the spinous layer cells, where γ-H2AX levels were greatly elevated, and green fluorescence was markedly enhanced. In the sample groups, as the PP-FP concentration increased, the green fluorescence in the nuclei decreased significantly, and γ-H2AX levels notably reduced, indicating a decrease in DNA damage in the cell nuclei. The HG-m group exhibited the best protective effect on the mice epidermis.

#### Regulation of inflammatory and MMP-related gene expression

**Fig. 9:**
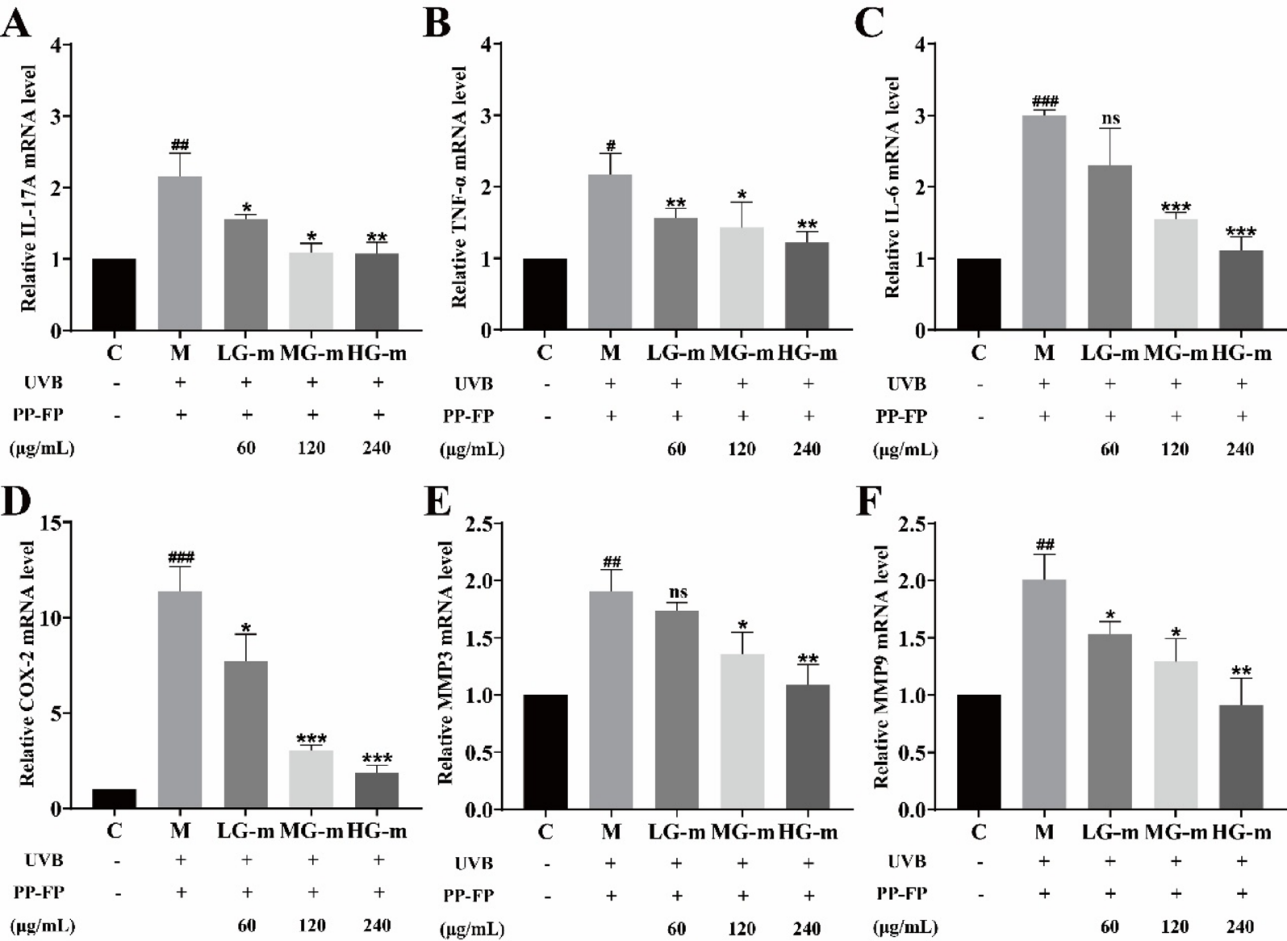
Effects of PP-FP on UVB-induced inflammatory and matrix metalloproteinase (MMP) gene expression in mouse skin. (# *p* < 0.05, ## *p* < 0.01, ### *p* < 0.001 compared with the control group; * *p* < 0.05, ** *p* < 0.01, *** *p* < 0.001 compared with the model group, ns indicates no significant difference compared with the model group.)

In vivo studies were performed to examine the impact of varying PP-FP doses on the levels of matrix metalloproteinases (MMPs) and inflammatory cytokines in murine dermal tissue. As shown in Fig. 9, experimental results indicate that UVB irradiation significantly upregulates MMP3 and MMP9 expression in mouse skin tissue, reaching 1.9-fold and 2.0-fold levels relative to the control group, respectively. This process also induces the release of numerous proinflammatory cytokines, including IL-17 A, TNF-α and IL-6, particularly cyclooxygenase-2 (COX-2).

Administration of varying doses of PP-FP resulted in a substantial suppression of both matrix metalloproteinases (MMPs) and these inflammatory markers within the murine dermal tissue. In particular, the transcriptional expression of IL-6 and COX-2 was profoundly lowered. In the high-dose treatment group (HG-m), gene expression of IL-17 A, TNF-α, IL-6, and COX-2 decreased by 50.12%, 43.88%, 62.88%, and 83.57%, respectively. These results demonstrate that PP-FP effectively inhibits UVB-induced inflammatory responses and matrix degradation at the transcriptional level in mouse skin.

## Discussion

The peel of *Passiflora edulis* Sims accounts for over 50% of the entire fruit’s weight and is the primary byproduct of the passion fruit processing industry. It is rich in natural active ingredients such as polysaccharides, polyphenols, and anthocyanins *(*Huo et al. 2023), and possesses potential biological activities including antioxidant and anti-inflammatory properties *(*da Costa et al. 2023). This study employed microbial fermentation technology, utilizing *Saccharomyces cerevisiae* to process *Passiflora edulis* Sims peel and extract fermented polysaccharides. The protective effect against UVB-induced skin photodamage was systematically evaluated, providing a new strategy for the high-value utilization of Passiflora edulis Sims peel waste.

Structural characterization results (Fig. 1) showed that PP-FP exhibited the typical functional group structure of polysaccharides, with a weight-average molecular weight of 6.66 kDa. It featured an intertwined fibrous structure with relatively uniform width and high roughness, conferring excellent rheological properties and adsorption capacity. The molecular weight of PP-FP was significantly reduced. The reported molecular weight of water-extracted passion fruit peel polysaccharides ranges from 10 to 50 kDa *(*Teng et al. 2022b; Li et al. 2021b), whereas the molecular weight of PP-FP in this study was only 6.66 kDa, indicating that the fermentation process effectively degraded the macromolecular polysaccharides. This phenomenon is consistent with other studies on fermented polysaccharides. Fang Long et al. *(*Long et al. 2026) reported that after fermentation with *Monascus purpureus*, the Mw decreased from 22.3 kDa to 8.84 kDa. Similarly, Mengyue Xu et al.*(*Xu et al. 2023) found that after fermentation with *Bacillus subtilis natto*, the molecular weight of lentinan was significantly reduced from 528.42 to 344.71 and 83.74 kDa. Low-molecular-weight polysaccharides possess better solubility and transdermal absorption capacity (Ro et al. 2020). Additionally, PP-FP contains a high proportion of moisturizing active ingredients, including glucose and galactose, making it a promising ingredient for skin hydration and functional regulation.

Research indicates that ultraviolet radiation leads to the accumulation of intracellular Ca²⁺and ROS, which serve as early markers of cellular damage. Excessive calcium influx can trigger cellular injury and apoptosis, while ROS accumulation disrupts the balance between oxidative and antioxidant processes, thereby inducing oxidative stress, mitochondrial dysfunction, apoptosis, and cellular inflammatory responses(Hsu et al. 2015; Baek et al. 2017). Therefore, this study established a UVB-induced damage model and further investigated the mechanism of action of PP-FP on HaCaT cells through in vitro experiments.

The results demonstrated that PP-FP enhanced the survival rate of UVB-damaged cells, maintained their migratory capacity, and effectively suppressed the accumulation of ROS and calcium ions within the cells (Fig. 3). Furthermore, PP-FP effectively mitigates the decline in mitochondrial membrane potential induced by UVB irradiation, manifested by a significant reduction in green fluorescence and an increase in red fluorescence. This indicates that PP-FP helps maintain normal mitochondrial membrane potential and reduces epidermal cell apoptosis (Fig. 4). Indeed, numerous studies have shown that plant polysaccharides possess excellent antioxidant activity*(*Li et al. 2026). Fermentation is capable of enhancing the biological activity of polysaccharides. Research indicates that after fermentation by *Saccharomyces cerevisiae*, the molecular structure of mushroom polysaccharides undergoes changes, significantly boosting their immunomodulatory effects. This process promotes the activation of immune cells and enhances immune function(Li et al. 2021a). Furthermore, the extracellular polysaccharide Cs-HK1b derived from Cordyceps sinensis exhibits enhanced anti-inflammatory activity following fermentation(Liu et al. 2019).

Additionally, UVB radiation induces an increase in proinflammatory factors (such as interleukins and tumor necrosis factor) induced by ultraviolet B radiation. PP-FP treatment inhibited these inflammatory factors in a dose-dependent manner. Notably, the 500 µg/mL group exhibited the most significant inhibitory effects on TNF-α and IL-8.

FLG (filaggrin), IVL (intercellular vesicle protein), and LOR (lamellar oligomerizing protein) are key structural and functional proteins constituting epidermal keratinocytes. IVL interacts with LOR to form a unique cross-linked structure—the keratin envelope—which serves as a defensive structure of the epidermal stratum spinosum, significantly contributing to its protective function(Zhang et al. 2023; Hashimoto-Hachiya et al. 2018). Aquaporin 3 (AQP3) is an integral membrane protein that mediates the passage of water and glycerol into the epidermis. This process bolsters the moisture-retention ability of the stratum corneum and supports the homeostasis of the skin barrier(Yi et al. 2022; Agren et al. 2010). MMPs (matrix metalloproteinases) are a class of are a class of enzymes capable of degrading collagen, leading to the breakdown of collagen and elastin in the skin. UVB irradiation resulted in decreased expression of FLG, LOR, IVL, and AQP3, along with increased activities of MMP3 and MMP9 in HaCaT cells. PP-FP treatment significantly ameliorated these alterations, leading to the upregulation of barrier protein expression and inhibition of MMPs activities.

This study demonstrated the protective effect of PP-FP on UVB-irradiated skin cells by measuring intracellular ROS, Ca^2+^ levels, mitochondrial function, inflammatory cytokines, and skin barrier-related proteins. Furthermore, external factors such as UVB irradiation can induce DNA double-strand breaks within cells. Immunofluorescence staining for γ-H2AX (phosphorylated H2AX) serves as an effective method for detecting DNA damage in cells or tissues(Mischo et al. 2005).

Excessive exposure to UVB radiation may trigger symptoms such as erythema, epidermal thickening, skin roughness, and accelerated wrinkle formation. In vivo animal studies further investigated the protective mechanisms of PP-FP against skin inflammation and barrier function impairment. Macroscopic observation revealed that the dorsal skin of mice in the model group exhibited severe redness, swelling, roughness, scaling, and wound formation. In contrast, the skin of PP-FP-treated mice, particularly in the high-concentration group (240 mg/mL), appeared smooth with only minimal scaling, closely resembling the control group. Hematoxylin and eosin (H&E) staining showed marked keratinization abnormalities in the dorsal skin of model group mice, whereas PP-FP treatment reduced epidermal thickness in a dose-dependent manner. Immunofluorescence staining for γ-H2AX revealed that the DNA damage marker was significantly increased in the epidermal and granular layers of the model group, indicating severe UVB-induced DNA double-strand breaks. PP-FP treatment significantly reduced γ-H2AX fluorescence intensity. Collectively, these findings demonstrate PP-FP’s exceptional protective efficacy against UVB-induced damage.

In recent years, an increasing number of plant polysaccharides have been reported to exert protective effects against UVB-induced photodamage*(*Long et al. 2023) demonstrated that *Dendrobium nobile* Lindl polysaccharides (DNP) alleviated epidermal thickening and collagen degradation in KM mice by activating antioxidant enzymes (SOD, CAT, GSH-Px), suppressing inflammatory cytokines (TNF-α, IL-1β, IL-6), and downregulating MMP-1/3/9 expression*(*Xu et al. 2024) reported that an exopolysaccharide (EPS) isolated from *Paenibacillus* sp. ameliorated erythema, edema, and collagen loss in C57BL/6 mice by scavenging ROS, restoring antioxidant enzyme activities, and downregulating IL-1β, IL-6, TNF-α, and MMP-3/9 expression*(*Neves et al. 2021) showed that a *Lycium barbarum* polysaccharide fraction (LBPF) combined with photobiomodulation (PBM) reduced epidermal thickening and collagen fiber fragmentation in hairless mice, but exhibited no significant inhibitory effect on MMP-1/2/9 expression. In comparison with the aforementioned studies, PP-FP in the present study demonstrated multi-target synergistic effects, combining antioxidant, anti-inflammatory, DNA protection, and barrier repair functions. Notably, PP-FP exerted its protective effects via topical application without the use of penetration enhancers, which is consistent with its low molecular weight (6.66 kDa) and porous structural features, facilitating its direct application in functional skincare products.

IL-17 is currently recognized as a critical signaling molecule in inducing inflammatory skin diseases such as psoriasis. It participates in regulating the secretion and synthesis of multiple inflammatory factors, including ILs and TNF-α(Chen et al. 2023b). Under normal conditions, IL-1β and its precursor IL-1α are present in healthy skin in a dormant state. Ultraviolet radiation promotes the activation of IL-17, which in turn activates the inflammasome protein NLRP3, leading to the activation of IL-1β and its precursor IL-1α. This induces pro-inflammatory interactions between keratinocytes and immune cells, thereby triggering inflammatory skin diseases(Yilmaz et al. 2012). Furthermore, excessive IL-17 secretion leads to abnormal proliferation and apoptosis of keratinocytes, with its immune-mediated skin inflammation involving NF-κB activation (Cho et al. 2012). The molecular-level analysis of this study focused on the regulatory effects of PP-FP on the expression of inflammation-related factors and matrix metalloproteinase (MMP) genes in the mouse epidermis. The results showed that PP-FP treatment significantly suppressed the UVB-induced upregulation of inflammatory cytokines and MMP gene expression. Compared with the model group, the high-concentration PP-FP treatment group (HG-m) exhibited reductions in the mRNA expression of IL-17 A, TNF-α, and COX-2 by 50.12%, 43.88%, and 83.57%, respectively, while the expression of MMP3 and MMP9 was also significantly downregulated. Notably, the inhibitory effects on COX-2 and IL-17 A were particularly pronounced in the high-concentration group.

IL-17, as a key driver of cutaneous inflammation, activates downstream adaptor proteins ACT1 and TRAF6 to initiate the NF-κB signaling pathway, thereby inducing the expression of multiple inflammatory cytokines and MMPs. Based on the regulatory effects of PP-FP on IL-17 A, TNF-α, and COX-2, it is speculated that its anti-inflammatory effects may involve modulation of the IL-17 signaling pathway. However, since the activation status of ACT1 and TRAF6 as well as the phosphorylation level of NF-κB were not directly examined in this study, the precise regulatory mechanisms remain to be further validated.

## Conclusion

As a by-product of *Passiflora edulis* Sims food processing, *Passiflora edulis* Sims peels are usually discarded and incinerated, causing great environmental pollution and resource waste, and *Passiflora edulis* Sims peels are rich in active ingredients. This study aimed to valorize the underutilized peels of *Passiflora edulis Sims* by extracting a polysaccharide (PP-FP) from its fermentation broth. The protective efficacy of PP-FP against UVB-induced damage was systematically evaluated through a combination of in vitro biochemical assays, cellular models, and in vivo experiments. The experimental results showed that PP-FP has a good photoprotective effect and has a high safety can be used safely as an efficacy component. This study not only provides a novel strategy for the high-value utilization of *Passiflora edulis* Sims peel a common agricultural byproduct but also highlights PP-FP as a promising natural-derived ingredient for skincare applications. Given the rising global prevalence of UV-induced skin damage and inflammatory dermatoses, PP-FP’s dual functionality in mitigating oxidative stress, inflammation, and barrier dysfunction positions it as a sustainable and multifunctional alternative to synthetic additives in cosmetics. Future research should focus on verifying whether PP-FP directly modulates the IL-17 signaling cascade, including the activation status of downstream adaptor proteins ACT1 and TRAF6, as well as the phosphorylation level of NF-κB. Additionally, its synergistic effects with other bioactive compounds or its efficacy in human clinical trials, Such investigations will contribute to a deeper understanding of the regulatory network underlying the protective effects of PP-FP and open new avenues for the development of eco-friendly functional skincare products.

## Data Availability

Data will be made available on request.
